# Health Consequences of the Female Genital Mutilation: A Systematic Review

**DOI:** 10.22086/gmj.v8i0.1336

**Published:** 2019-01-01

**Authors:** Khadijeh Sarayloo, Robab Latifnejad Roudsari, Amy Elhadi

**Affiliations:** ^1^Department of Midwifery, School of Nursing and Midwifery, Mashhad University of Medical Sciences, Mashhad, Iran; ^2^Nursing and Midwifery Care Research Centre, Mashhad University of Medical Sciences, Mashhad, Iran; ^3^College of Food, Agricultural, and Environmental Sciences, Program Development & Evaluation, Ohio State University, Columbus, Ohio Area, United States

**Keywords:** Female genital, Female, Obstetric Labor Complications, Pregnancy Complications, Emotional Aspects

## Abstract

Female genital mutilation (FGM) is a general health concern. The World Health Organization has recognized it as a condition that endangers women’s health. This review study aimed to identify the types of health outcomes of FGM. Therefore, a systematic review was conducted to create a critical view of the current evidence on the effect of Female genital on girls and women’s health. In this study, we focused on the health risks of female Female genital. Academic databases such as PubMed, Science Direct, Scopus, Google Scholar, Cochrane Database of Systematic Reviews, SID, IranMedex, Irandoc, and Magiran were searched with regard to the health consequences of FGM from January 1990 until 2018. Eleven review studies met the criteria and contained 288 relevant studies on the risks of FGM. It was suggested that FGM had various physical, obstetric, sexual, and psychological consequences. Women with FGM experienced mental disturbances (e.g., psychiatric diagnoses, anxiety, somatization, phobia, and low self-esteem) than other women. Our study can provide evidence on improving, changing behaviors, and making decisions on the quality of services offered to women suffering from FGM.

## Introduction

Female genital mutilation (FGM) can lead to injuries in genitalia organs, because of non-medical causes and has health disadvantages for women. Unprofessional and traditional circumcisers may damage the external sexual organs, but it may happen in 18% of cases in health care systems. An increasing trend of damage to the external sexual organ has been reported [1]. FGM is a popular procedure around the world including Africa (27 countries), Asia, North-America, and Europe. It has been reported that 125 million women and girls in the world undertake Female genital[2]. Media significantly advertise FGM in African and capitalist countries as a policy to revert sexual desire and woman’s personality, integrity and importance. Improvement of sexual quality, better appearance, and relieving pain among women undergoing FGM has been supported by the national health insurance in France [3]. The World Health Organization (WHO has provided a classification of the FGM (Table-1) [4]. The most severe type of FGM is called infibulations that happens in approximately 15% of all women with FGM [5]. Also, FGM could damages normal sexual organs in women and disrupt the normal function of sexual organs. FGM has some immediate and long-term risks. Acute pain, shock, bleeding, tetanus, septicemia, gangrene, HIV, hepatitis B and C, ulcers in genital organs, and deaths are some immediate outcomes [1]. The consequences of FGM are summarized in three major sections as obstetrical, gynecological, and psychological (especially sexual activity). The long-term consequences of FGM are described as chronic vaginal and pelvic infections, painful sexual intercourse, scarring, recurrent cystitis, urethritis, menstrual disorders, infertility, and psychological damages (such as low libido, depression, and anxiety). It may also increase the risk of pregnancy complications, neonatal mortality, and future surgeries [1, 6]. Sometimes, they need to undertake consecutive procedures such as stitching for several times after childbirth, which increase immediate and long-term risks [1, 6, 7]. Infection commonly occurs in FGM type III. Therefore, it is believed that FGM is a risk factor for genital disorders [8]. A study in six African countries showed that FGM increased the risk of complications in labor and childbirth [9]. Before designing an intervention to improve men and women’s knowledge about how FGM can be reduced, it has become progressively common, and various studies in other contexts have been conducted on it. Systematic review studies have been carried out on FGM and have found some problems related to FGM, but they failed to explain all related consequences. The emphasis of the WHO is zero tolerance for FGM. Systematic reviews are the best forms of research evidence [10]. There are many systematic reviews on the health consequences of FGM. Therefore, a synthesis of systematic review studies related to the health consequences of FGM was conducted to prepare a critical view of the scientific issues of FGM on related physical, psychological, social, and sexual consequences.

## Search Strategies

**Figure 1:**
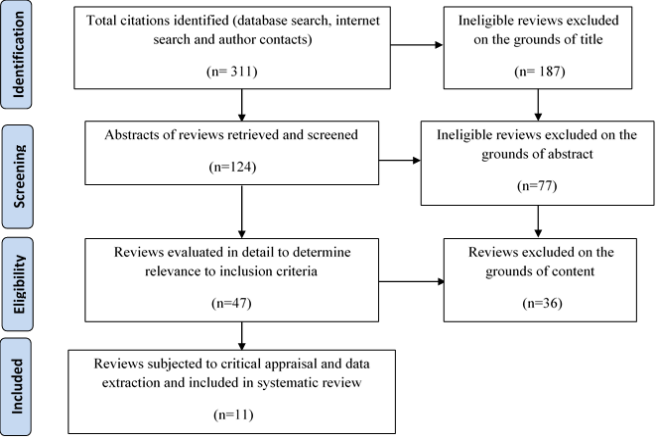
Flow diagram of the selected review.

A comprehensive search was performed in databases such as PubMed, Cochrane, Science Direct, Scopus, Google Scholar, SID, IranMedex, Irandoc, and Magiran. Other sources such as “gray literature” or non-electronic journals were not considered. Keywords used in the search process were as follow: ‘Female genital, ‘female,’ ‘complications,’ ‘FGM,’ ‘obstetric labor,’ ‘pregnancy complications,’ ‘emotional aspects’ and ‘review’ from January 1990 up to 2018. After the primary search, the second search processes were conducted based on the findings of the primary search. Ultimately, reference checking was carried out through a manual search to recognize any other relevant systematic reviews. The full-texts of all identified studies were retrieved and reviewed. Those systematic reviews that evaluated the health consequences of FGM were included in this study. Integrative, narrative or traditional reviews and updates, those reviews that focused on non-health consequences of FGM and published in non-English languages were excluded. Titles and abstracts of the studies were retrieved and assessed for inclusion by two reviewers, independently. Differences were resolved through discussions (Figure-1). The aim, search process, eligibility criteria, the total number of included studies, method of quality assessment, synthesis method, outcomes of FGM, conclusions, and recommendations were extracted from the studies and recorded in a predetermined form. All systematic reviews were critically appraised using the Database of Abstracts of Reviews of Effects (DARE) checklist [11, 12]. This checklist highlighted differences, reliability, and validity of the selected studies (Table-2).

## Results

According to our search process, 311 studies were related to our study’s aim, and 11 reviews had required eligibility criteria. Three of the studies were related to physical health outcomes after FGM [7, 11, 12] and three others included sexual consequences in women undergoing FGM [13-15]. Three reviews addressed the obstetric consequences of FGM including antenatal sequelae, labor and delivery, and childbirth sequelae [16-18]. One study addressed the psychological consequences of FGM including somatization disorders, psychological disorders, low self-confidence and nervousness [13]. Another study discussed FGM and fistulaas obstetric labor, and pregnancy complications [19] and the last one addressed economic and social impacts of FGM [20]. The results were summarized in Tables-3 and 4, and a narrative synthesis was also presented below.

### 1. Physical and Sexual Health Consequences of FGM

#### 1.1. Physical Health Consequences

FGM had some health outcomes, but no statistically significant report of the number of health situations was found. Infection such as acute infections after trauma, infection in the urogenital system, septicemia or HIV were associated with FGM type III [7]. A review study reported microbial agents in women after FGM based on 21 studies. FGM was conducted by physicians, paramedical staff, midwives, traditional healers, barbers, older women, and family members. It was stated the variety of infections occurred after FGM. Viruses, bacteria, parasites including HIV, Clostridium, Chlamydia, Neisseria, *Treponema pallidum*, Candida, *Trichomona svaginalis* were detected, and the risk of infection was reported as 0.47% to 5.2 % [7]. Another review [11] on 185 studies presented the main systemic risks of FGM over life. The most common immediate complications were bleeding (5–62%), edema in genitalia (2–27%), urinary infectious (8–53%), which were reported in women with FGM based on observational studies. In general, more than one urgent complication was found in every one of them. The probability of immediate damages was higher in type III than types I–II. The comparative studies reported genital infectious, long-term urogenital complications and menstrual disorders. A higher risk of urogenital infections in women with FGM was reported by ten comparative studies [11].

#### 1.2. Sexual Consequences

Four reviews focused on the sexual outcomes of FGM [13-15, 20]. One review study based on four studies reported a variation of sexual outcomes such as the form and function of the clitoris, vulvar danio, sexual activity and orgasm [15]. The studies poorly reported clitoral surgeries. The largest cohort study in France surveyed the effect of surgical reconstruction after FGM in women and showed a 46% low progression in clitoral pleasure, and 51% limited or normal orgasms [15]. In the other cohort studies [15], 21% had a normal clitoris after a 6-month follow-up. All these studies have limitations regarding follow-up time and the use of valid instruments to assess clitoris. Pain unrelated to coitus was reported in 3% of cases, and moderate-to-severe pain during coitus was available in 24% of cases in the largest cohort study [15]. There was a weak report of clitoral improvements after surgery in women with Female genital; therefore, the authors suggested that vigorous evidence on the safety and efficacy of Female genital was needed. The effect of sexual therapy and education on the improvement of sexual outcomes was not evaluated in the studies [15]. Another review and meta-analysis study by Berg *et al*. [14] assessed the sexual outcomes of FGM based on the reports of 15 studies. Painful coitus (>1.5 times) and low sexual satisfaction were more likely reported in women with FGM. The adverse outcomes of sexual function after FGM were reported including low sexual satisfaction and lack of desire. In a meta-analysis, scars and infections in most women with FGM type I or II and severe pain in type III due to mechanical obstruction were reported. More studies were needed to assess the health outcomes of FGM in women [14]. Berg *et al*. (2010) conducted a systematic review and examined the sexual consequences of FGM [13]. All studies except two reviews showed that painful coitus, low satisfaction, and low desire were more likely in FGM. Painful coitus were 1.5 times more likely (relative risk [RR]=1.52, 95% confidence interval [CI]=1.15, 2.0) and no sexual desire were twice more likely in FGM (RR=2.15, 95%CI=1.37, 3.36). Berg *et al*. [13] found that dyspareunia, low satisfaction, and desire were more likely in women with FGM, but the low-quality of the study prevented to take a decisive conclusion. Sexual desire, arousal and psychological issues such as anxiety were more common problems in these studies. Fifteen studies reported that the majority (58.5%) of sexual outcomes were statistically associated with the level of literacy. A significant number of women with FGM reported sexual disorders including dryness in coitus, dyspareunia, no sexual pleasure and no orgasm in several studies. Finally, the meta-analyses concluded satisfaction, desire, initiation of sex, orgasm as the sexual outcomes of FGM [13]. The health and sexual risks of FGM in women were explored using a review study by Obermeyer *et al*. [12] Those studies compared circumcised women with others and indicated that they were at the higher risk of anemia and infection. For instance, bacterial vaginosis and herpes simplex virus 2 (HSV2) were more common in women with FGM. Another trial in Nigeria demonstrated that pain in the abdomen, discharge, and sores in genitalia were more prevalent in women with FGM [12]. The frequency of bleeding and infections were reported in wide ranges. For example, swelling and edema were reported in 2-50% and urine retention in 12-70% of cases. A higher incidence of cysts, perineal scarring, and problems in relation to anatomical damage were reported. None of the studies provided the estimation of increased risks, but they suggested a higher chance of health-related problems. Some studies indicated a higher risk of perineal laceration, fetal distress, and general difficulties. There was a high gynecological effect including abdominal pain, discharge, and ulcer [12]. Mpinga *et al*. (2016), reported sexual and marital issues as the socio-economic consequence of FGM in women [20]. They reviewed 198 articles, but only seven studies (3.5%) focused on the sexual consequences and marital problems of FGM. Marital/sexual disorders (i.e., dyspareunia, loss of libido, failure of orgasm, and husband’s dissatisfaction) were higher among women with FGM. Some sexual disorders were reported by circumcised women such as painful coitus (31.5%), low sexual desire (49.6%), the problem with arousal (36%), and lack of orgasm (16.9%). Circumcised women reported pain and dryness during coitus (48.5%),sexual arousal disorders, e.g. lack of desire (45%), reduced frequency of desire (28%), low pleasure (49%) and lack of orgasm (39%), and difficulty reaching the orgasm (60.5%) [20].

### 2. Obstetric Health Consequences of FGM

Four reviews [16, 18-20] reported the obstetric impacts of FGM. One systematic review [17] and meta-analysis provided clear evidence that FGM significantly increased the risk of childbirth complications. In this study, some obstetric outcomes of FGM such as prolonged or difficult labor, laceration, cesarean, episiotomy, instrumental delivery, hemorrhage were assessed, but the risk of prolonged labor, laceration in the perineum, and hemorrhage were significantly higher than others. Only two prospective studies [17] with 28 studies (about 20,000 women) showed a statistically significant result with no heterogeneity. The risk of cesarean section (odds ratio [OR]=1.60) was higher. The results explained a (borderline) statistically significant result of harm from FGM, (𝐼-squared=96%). There were inconsistent findings about instrumental delivery, but a considerable heterogeneity by studies was reported (𝐼-squared=91%), and neither harm nor benefit could be ruled out. Out of eight studies (746,667 women), which gave a pooled OR of 2.18 (95% CI=1.40, 3.37), a greater risk of postpartum hemorrhage (𝐼-squared=93%) was reported. Significantly, increased risk of the complications of labor and delivery in FGM was reported [17]. Berg *et al*. (2013) conducted a review and meta-analysis and assessed 44 primary studies [13]. The prolonged labor, laceration, hemorrhage, instrumental delivery, and difficult delivery were significantly associated with FGM indicated that Female genital increased the risk of complications of labor and delivery. Nine of the studies [13] assessed prolonged labor in women in two groups (with and without FGM). The absolute risk was more than three times in the FGM group (95% CI=0–8). Therefore, significant differences were reported between them especially for the non-FGM group (RR=1.69). A remarkable effect of obstetric lacerations was found (RR=1.38) with an absolute risk for more than 1.5 times in the FGM group. Also, the absolute risk difference was reported five more cases of obstetric hemorrhage and difficult labor in the FGM group. No statistically significant effect of cesarean and episiotomy was found. It showed that the deliveries of the FGM group were more likely to be complicated compared to other groups [16]. In another systematic review, 65 studies of a total of 422 studies reported the different types of health consequences as follow: obstetrical dysfunction in antenatal, pregnancy, labor, delivery and postpartum, maternal death and neonatal fatality. Gynecological disorders consisted of menstrual, urogenital problems, and immediate problems following FGM. Also, pregnancy associated with pinhole introitus (eight studies), horror from childbirth and need for special care (five studies), difficulty in vaginal examinations (six studies), urinary retention in labor (four studies), difficulty in assessing progress in labor by the vaginal examination (seven studies), prolonged labor and/or obstruction as one of the most frequent obstetric outcomes of FGM (29 studies), fetal distress (four studies) and pain during and after de-Female genital (4 studies) were reported. WHO report (2000) [18] showed that postpartum hemorrhage was remarkably more common in women with FGM types I, II or III. Additional incisions and perineal lacerations as a result of the scarring from FGM were reasons for severe hemorrhage. Seven studies identified maternal death following FGM happening earlier in life. Four of the ten studies reported stillbirth and neonatal deaths. Genital ulcer infection was recognized after childbirth as a complication of FGM, and the rate of infection was higher in the wound caused by FGM type III compared to type I. In seven studies, fistulae and postpartum hemorrhage were other complications especially in type III. Outcomes such as a painful scar, preterm labor, obstruction (vaginal atresia) requiring caesarean section, difficult labor, neonatal death, antenatal problems to make difficulty in the vaginal examination, vaginal atresia in pregnancy, and antenatal fetal injury were identified by one study. Also, maternal mortality, possible antenatal vesicovaginal and/or rectovaginal fistula and fetal death following FGM were recognized by two studies [18]. In Mpinga *et al*. review [20], out of 198 articles, only five studies explored fertility as the socio-economic consequences of FGM in women and all of them except one study reported a link between FGM and infertility. One study reported that the infertility rate could be as high as 30% in infibulated women and associated with FGM type III [20]. Pooja *et al*. (2017) conducted a review to study the association between FGM and fistula [19]. Out of the 30 studies in their review, eight studies positively were related to the association between FGM and the occurrence of fistula, but three studies have not any relationship, and 18 studies found an indirect positive relationship with various degrees of evidence. In 19 studies, the association between FGM and the fistula was reported. Other studies reported indirect mechanisms through, which FGM affected fistula in relation to health consequences including those that might occur at childbirth, and get moderated by the levels of clinical management. Some studies found that at the time of, and immediately following, cutting procedures (particularly infibulation), women were at the risk of urinary, and traumatic fistula [19].

### 3. Psychosocial Consequences of FGM

According to the systematic review and meta-analyses by Berg *et al*. (2010) [13], 17 comparative studies evaluated the psychological consequences of FGM. Four of the studies measured post-traumatic stress disorder (PTSD) and general psychiatric symptoms including depression, anxiety, and phobia. Psychological disorders such as having a psychiatric diagnosis, suffering from anxiety, somatization, phobia, and low self-confidence might be more likely in FGM group than other groups. The meta-analyses [13] failed to provide evidence for anxiety, somatization, depression, and hostility in women with FGM. Also, psychological problems were not fully described. Only two studies measured the social outcomes of FGM but had low levels of quality for making appropriate conclusions. Two studies estimated PTSD, and one of them used the PTSD inventory, and another applied the short, structured diagnostic interview. Two studies reported the continuous outcomes of psychological consequences. Only one study found a significant difference between PTSD and FGM. Another study found that psychological disturbances such as levels of anxiety, somatization, and phobia were significantly higher in women with FGM compared to others. Only two low-quality studies showed the high levels of marital dissatisfaction in the FGM group (43.0%) compared to others (10.9%). Also, marital instability was higher in FGM than others. The meta-analyses revealed that no statistically significant effect of anxiety, somatization, depression, and hostility [13].

## Discussion

This study assessed the outcomes of FGM based on the report of 311 studies of the consequences of FGM. Overall, it was found that FGM had complications in terms of physical, obstetrical, sexual and psychosocial outcomes [7, 11-18].

### Physical and Sexual Consequences

Different types of infections were reported including acute local trauma infections, urogenital infections, abscess, and septicemia or even HIV, especially in type III of FGM. This finding was supported by a previous study [7]. There was a greater risk of long-term outcomes of FGM such as urinary tract infection, bacterial vaginosis, painful coitus, and obstetrical problems. Long-term outcomes of FGM are vigorous, and the least increase of such problems has a negative effect on women health [17, 20]. The most common immediate complications of FGM are excessive bleeding, urine retention, swelling and edema in genitalia, problems with wound healing, and pain [11]. There is a significantly more significant proportion of women with FGM who complained of negative sexual experiences such as vaginal dryness during coitus, dyspareunia, sexual relationship without pleasure, anorgasmia, the dissatisfaction of sexuality and delay in the initiation of sex [13]. The more rigorous evidence is needed on the safety and efficacy of FGM. The impact of sexual therapy and education were not evaluated on alleviating pain or improving sexual outcomes [15]. The review confirmed that marriage and sexual dysfunction such as painful intercourse, loss of sexual urge anorgasmia, lack of sexual desire were associated with FGM [20].

### Obstetric Consequences

Prolonged or difficult labor, lacerations, cesarean, episiotomy, instrumental and complicated delivery, hemorrhage in the form of direct bleeding, procedure-related complication, most likely due to the rupture of the internal pudendal artery or the clitoral artery are reported as the obstetric outcomes [17]. The results obtained from reviews indicate that FGM was an essential factor in the occurrence of complications of childbirth and significantly increased the risk of complications [17]. In a systematic review, no significant effect of FGM on cesarean section and episiotomy was reported [16]. WHO categorized the immediate outcomes of FGM as obstetric (antenatal, labor, delivery, postpartum hemorrhage, pregnancy complications, maternal mortality, and neonatal mortality, fetal distress, stillbirth), gynecological (menstrual problems), and urinary problems. Maternal death may have happened earlier in life [18].

### Psychosocial Consequences

Studies have shown that FGM has many psychological implications including psychiatric diagnosis anxiety, somatization disorders, phobia, and low self-esteem. Furthermore, another study found various emotional difficulties including loss of trust between mother-daughter, feeling of fear, helplessness, and anger. Due to the low-quality of studies, the relationship between FGM and psychological consequences could not be confirmed [13]. This systematic reviews showed that several physical outcomes always followed FGM. Therefore, it endangers women’s health, and women should be informed about the scarcity of evidence related to improved outcomes. Comprehensive education is needed on how sex therapy and surgery can improve sexual activity and body image. Midwives and healthcare providers should receive education to educate women about FGM including its relationship with sexuality and obstetric issues. Also, FGM is a sensitive issue and taboo in societies, considering that facilities are required for professional communication and management. The strengths of this review were that the researchers read all available systematic reviews on FGM and its health consequences. It emphasized review studies rather than investigating individual studies. Accordingly, evidence suggests that FGM has adverse outcomes and problems for women’s health throughout life including sexual activity and delivery. As a limitation, there was a different level of reports in the original reviews. Also, some studies had no quality appraisal and assessment sections. Our review complied with DARE criteria for systematic reviewspublished until 2018, but newly published reviews could be available. Also, we could not access the full-text of three relevant studies despite extensive retrieval efforts. The DARE checklist was used to assess the quality of the reviews for all outcomes to confirm conclusions about a causal connection between FGM and health complications. Since there were no standardized definitions for measuring common outcomes, the assessment of prolonged labor, and sexual functions.

## Conclusion

This study showed the presence of the severity of injury among women with FGM compared to non-circumcised women. There are sufficient reasons to conclude that FGM carries physical, sexual, obstetric, and psychological damages to women health. Irrespective of the size of the risk of FGM, increased obstetric complications, and the morbidities can justify the cessation of the practice. The improvement of the women’s socio-cultural status in combination with planning programs to enhance their information and awareness as well as trying to change the cultural leaders’ viewpoints regarding this procedure is essential to reduce FGM and its burden on women health.

## Conflicts of Interest

The authors declare no conflicts of interests.

**Table 1:** The WHO Classification of FGM [4]

**Type I**	Partial or total removal of the clitoris (clitoridectomy) and/or the prepuce
Ia	Removal of the prepuce/clitoral hood (Female genital)
Ib	Removal of the clitoris with the prepuce (clitoridectomy)
**Type II**	Partial or total removal of the clitoris and the labia minora, with or without excision of the labia majora (excision)
IIa	Removal of the labia minora only
IIb	Partial or total removal of the clitoris and the labia minora
IIc	Partial or total removal of the clitoris, the labia minora and the labia majora
**Type III**	Narrowing of the vaginal orifice with the creation of a covering seal by cutting and appositioning the labia minora and/or the labia majora, with or without excision of the clitoris (infibulation)
IIIa	Removal and appositioning the labia minora with or without excision of the clitoris
IIIb	Removal and appositioning the labia majora with or without excision of the clitoris
**Type IV or** **Unclassified**	All other harmful procedures to the female genitalia for non-medical purposes, for example: pricking, pulling, piercing, incising, scraping and cauterization

**Table 2:** Database of Abstracts of Reviews of Effects (DARE) Checklist

1. Is there a well-defined question?
2. Is there a defined search strategy?
3. Are inclusion/exclusion criteria stated?
4. Are the primary study designs and number of studies clearly stated?
5. Have the primary studies been quality assessed?
6. Have the studies been appropriately synthesized?
7. Has more than one author been involved at each stage of the review process?

**Table 3:** Summary of the Systematic Reviews

**Author, Year**	**Review objective**	**Review inclusion criteria**	**Population**	**Study design**	**Location**	**Databases searched**	**Relevant primary study Number**	**Total number of included studies**	**Synthesis method**	**Quality of included studies**	**Ref.**
Abdulcadir *et al*., 2015	To review evidence on the safety and efficacy of clitoral reconstruction	Studies of any design that reported safety or clinical outcomes (e.g., appearance, pain, sexual response, or patient satisfaction), associated with clitoral reconstruction after FGM	Individuals with clitoral reconstruction after FGM	Case-control=3 Prospective cohort=3	Switzerland	PubMed, Cochrane databases	269	4	Narrative	II-2 Poor= 1 II-3 Poor=3	[15]
Berg *et al*., 2013	To clarify the present state of empirical research	Studies providing quantitative data on physical consequences with any study design, except qualitative studies, study design features	Women that were subjected to any type of FGM, and the exposed to FGM, classified as type I to IV according to the WHO modified typology	Comparative studies=21 Single group cross-sectional studies=7 Case series=5 Case reports=4	Norway	MEDLINE, African Index Medicus, British Nursing Index and Archive, CINAHL, the Cochrane Library, EMBASE, PILOTS, POPLINE, PsycINFO, Social Services Abstracts, Sociological Abstracts, and WHOLIS	5109	44	Statistical pooling	Low=29 Moderate= 5 High=6 Not applicable=4	[16]
Berg *et al*., 2014	To validate the results through additional analyses based on adjusted estimates from prospective studies	28 studies for prospective features, that is whether the women’s FGM status was assessed before delivery	Women’s FGM status was assessed before delivery	Prospective=7 Observational=21	Norway	The Cochrane Handbook for Systematic Reviews of Interventions	Not provided	28	Statistical pooling	Low and very low	[17]
Iavazzo *et al*., 2013	To explore and analyze the clinical evidence related to the presence of infections in the practice of FGM	Studies reporting data on infection related to patients with FGM	Patients with FGM	Retrospective Study=2 Prospective Study=2 Survey=11 Case-control =1 Cross-sectional =3 Case report=2	Greece	PubMed and Scopus	1078	21	Narrative	Unclear	[7]
Berg *et al*., 2011	To conduct a systematic review and meta-analysis of the sexual consequences of FGM	Women with FGM 9classified as types I–IV according to the WHO modified typology) and women without FGM	Women with FGM	Cross-sectional comparative (clinical-/ hospital-based) and =8 Prospective case–control=1 Purposive sampling=2 Cross-sectional comparative (community-based) =1 Cross-sectional comparative (cluster sampling) =1 Cross-sectional comparative (subsample of DHS) = 1 Case-control (clinical-/ hospital-based)=1	Norway	African Index Medicus, Anthropology Plus, British Nursing Index and Archive, The Cochrane Library, , EMBASE, EPOC, MEDLINE, PILOTS, POPLINE, PsycINFO, Social Services Abstracts, Sociological Abstracts, and WHOLIS	7515	15	Statistical pooling	High=2 Moderate =3 Low=10	[14]
Berg *et al*.,2015	To systematically review evidence for physical health risks associated with FGM	Empirical studies reporting physical health outcomes from FGM	Women with FGM	Case-control studies=3 Cross-sectional=30 Prospective=7 Based on the DHS=9 Retrospective cohort=1 Registry study=6 Unclear if prospective or Retrospective=1	Norway	The Cochrane Handbook for Systematic Reviews of Interventions, MEDLINE, Open Grey, Open Sigle, and OAIster	5109	57	Statistical pooling	High=10 Moderate=19 Low=28	[11]
Obermeyer *et al*.,2005	To provide a careful assessment of the evidence and suggest ways to avoid the pitfalls of research on the subject	The main criterion was whether the source provided new data on the association between female Female genital and health and sexuality effects	women who exposed and unexposed to FGM	Randomized controlled trials following an experimental design Prospective cohort studies	USA	MEDLINE and Sociofile	500	35	Narrative	Good	[12]
Berg *et al*., 2010	To conduct a systematic review of the consequences of FGM	Reviews and studies on experts engaged in FGM related work	Women with FGM experience pain and reduction in sexual satisfaction	Cross-sectional studies=15 case-control studies=2	Norway	African Index Medicus, Anthropology Plus, British Nursing Index and Archive, The Cochrane Library (CENTRAL, Cochrane Database of Systematic Reviews, Database of Abstracts of Reviews of Effects), EMBASE, EPOC, MEDLINE, PILOTS, POPLINE, PsycINFO, Social Services Abstracts, Sociological Abstracts, and WHOLIS.	4434	17	Statistical pooling	Low=10 Moderate=5 High =2	[13]
WHO report, 2000	To identify primary data on health complications of FGM, with a particular emphasis on sequelae in childbirth, including psychosexual outcomes.	Original articles (or, exceptionally, a review of unpublished/not yet obtained data) in any language about humans _ about health complications of FGM or topics that are important to the health complications of FGM (e.g., vaginal atresia)	Antenatal complications and complications in early labor in women with FGM	Original data review papers and news original data was sub-divided into cases and case series, and comparative studies with groups compared or with cases and controls.	WHO Genena	MEDLINE EMBASE CINAHL Psychlit Sociofile SIGLE CAB Health ExtraMed Popline AHTRAG/HealthLink Hand search of key journals	422	67	Narrative	Unclear	[18]
Mpinga *et al*., 2016	To characterize over a 40-year period the scientific output on the consequences of FGM in African countries, the most affected region known for the high prevalence of FGM, and review data on the socioeconomic consequences of the procedure	Articles concerning the consequences of FGM	Women who had undergone any type of FGM	Cross-sectional=64 Cohort=16 Case-control=4 Qualitative studies=10 Case series=14 Social analyses=40 Economic studies=3 Simple reviews=28 Systematic reviews=2 Other (educational recommendations, reports of conferences) =17	Switzerland	PubMed, EMBASE, CINAHL, BDSP, Web of Science, PsycINFO, FRANCIS, Sociological Abstracts, WHOLIS, RERO, and SAPHIR	2470	198	Narrative	Unclear	[20]
Sripad *et al*., 2017	1) assess the state of evidence on the association between FGM and fistula, 2) conceptually map this association within broader social, political, and health systems contexts, 3) identify evidence gaps and areas for further research, and 4) develop recommendations for policy and programming.	Studies were selected for inclusion in the body of evidence exploring the association between FGM and fistula	Women who had undergone FGM and nurses, midwives, surgeons, and obstetricians, gynecologists, policymakers, opinion leaders, and community members	Observational =18 Systematic review=4 Other type =8	UK	PubMed, Google Scholar, Scopus, JSTOR, Brandeis Scholar, Population Council, UNFPA, and Engender Health	512	30	Narrative	High=40% Medium =43% Low =17%	[19]

**Table 4:** Main Findings of the Systematic Reviews

	**Health outcome**	**Main findings**	**Ref.**
Abdulcadir *et al*., 2015	Safety or clinical outcomes (e.g., appearance, pain, sexual response, or patient satisfaction) associated with clitoral reconstruction after FGM	The case-control study did not report safety, postoperative clitoral appearance, chronic pain/dyspareunia. Prospective cohort reported complications, chronic pain/dyspareunia: pain without sexual intercourse and moderate-to-severe dyspareunia preoperatively chronic pain/dyspareunia, moderate-to-severe pain with sexual intercourse, restricted or regular” orgasm.	[15]
Berg *et al*., 2013	Obstetric consequences of FGM	9 studies reported 3 more cases of prolonged labor among women with FGM. 15 studies reported obstetric tears/lacerations among women with FGM. 15 studies reported no statistically significant difference for cesarean section. 11 studies reported no significant effects for an episiotomy. 3 studies reported instrumental delivery. 9 studies reported obstetric/postpartum hemorrhage. 7 studies reported difficult labor/dystocia. 7 studies reported difficult labor/dystocia.	[16]
Berg *et al*.,2014	FGM and obstetric complications	All studies reported women with FGM are at significantly higher risk of experiencing prolonged labor. 5 studies indicated both harms and benefited from FGM. All studies showed women with FGM were at a significantly higher risk of experiencing difficulties during delivery. There is uncertainty about the size of the greater obstetric risk of harm among women with FGM, sufficient grounds to conclude that FGM involves obstetric complications.	[17]
Iavazzo *et al*.,2013	The infections related to patients with FGM including UTIs, genitourinary tract infections, abscess formation, and septicemia or even HIV infection	A variety of infections can occur after FGM. The management of these complications in a low-income economy can be a high burden on families. 2 studies reported AIDS after FGM. 1 studies reported STD after FGM. 5 studies reported acute local infection after FGM. 3 studies reported urinary tract infection after FGM. 1 studies reported STD, AIDS, reproductive tract infections after FGM. 1 studies reported genitourinary tract infections after FGM. 1 studies reported reproductive tract infections after FGM. 1 studies reported localized infection/ abscess, septicemia after FGM. 1 studies reported endogenous infections STD after FGM. 1 studies reported urinary tract infections, vaginal infections after FGM. 1 studies reported puerperal sepsis from infected Female genital scars after FGM.	[7]
Berg *et al*.,2011	Sexual consequences of FGM	A woman who her genital tissues have been partly removed is more likely to experience increased pain and reduction in sexual satisfaction and desire. FGM of any type may be associated with sexual problems	[14]
Berg *et al*.,2015	Physical health outcomes from FGM, menstrual complications, obstetric complications, vaginal calculus formation, cysts, tissue injury, fractured/displaced bones, meatal urethral stenosis/ urethral stricture, abscesses, keloid and another scarring (secondary outcomes).	The most common immediate complications were excessive bleeding, urine retention, and genital tissue swelling. The most valid and statistically significant associations for the physical health sequelae of FGM were seen on urinary tract infections, bacterial vaginosis, dyspareunia, prolonged labor, cesarean section, and difficult delivery. 56 observational studies reported on eight main types of immediate medical harms (bleeding, shock, genital tissue swelling, fever, infections and problems with urination and wound healing) on females and types of FGM. 3 clinical reports on deaths directly attributed to FGM. 4 cross-sectional studies reported a higher risk of vaginal discharge and itching with FGM 4 comparative studies reported long-term urological complications. 5 studies reported menstrual problems. 10 comparative studies cited results concerning long-term genitourinary infections. 6 studies indicated dyspareunia (painful sexual intercourse). 26 comparative reported obstetric events (prolonged labor, tears/lacerations, cesarean section, episiotomy, instrumental delivery, hemorrhage, difficult labor). 6 studies reported prolonged labor.	[11]
Obermeyer *et al*.,2005	Health consequences, Urinary problems, Infertility, labor and delivery problems, pain, dysmenorrhea, sexual problems, and sexual behaviors	1 study showed bacterial vaginosis and HSV2 were more frequent among circumcised women 1 study found abdominal pain, discharge and genital ulcers more frequent among circumcised women. The studies reported very wide ranges in the numbers reported for bleeding and infections. Carefully controlled studies did not find a statistically significant increase in infertility. Studies of labor and delivery problems indicate significantly higher risks of self-reported perineal tears, fetal distress, and general difficulties. Controlled studies reported increased risks of reporting abdominal pain, discharge and ulcers among circumcised women, and general gynecological problems. There is a growing body of evidence on the health consequences of female genital cutting, and that a more diverse set of complications are now included in the research. The study reported statistically higher risks for some but not all types of infections; urinary symptoms; obstetric and gynecological complications: increased risks have been reported for some complications of labor and delivery but not others, and for some symptoms such as abdominal pain and discharge, but not others such as infertility or increased mortality of mother or infant. The studies do not support the hypotheses that Female genital destroys sexual function or precludes the enjoyment of sexual relationships.	[12]
Berg *et al*.,2010	Psychological, social and sexual consequences	The studies reported pain during intercourse, reduced sexual satisfaction, and decreased sexual desire. 15 studies reported significant differences in sexual consequences including satisfaction during intercourse, arousal, lubrication, orgasm, satisfaction, sexual excitement, sexual problems, experiencing painful sex, dyspareunia, initiating sex and most sensitive bodily part. 4 studies reported psychological, social outcomes including somatization, anxiety, phobia; marital satisfaction. 4 studies reported self-esteem; marital instability, post-traumatic stress disorder, affective disorders, psychiatric diagnosis.	[13]
WHO report, 2000	Obstetric sequelae of FGM, antenatal sequelae, prolonged labor and/or obstruction, pain during and after de-Female genital, post-partum hemorrhage	8 studies reported pregnancy in the presence of pinhole introits 5 studies showed fear of labor and delivery due to the small size of introits and needed for appropriate obstetric care. 6 studies indicated difficulty in performing antenatal vaginal examinations. 1 study reported painful scar. 4 studies reported urine retention in labor. 7 studies identified difficulty in assessing progress in labor by vaginal examination. 29 studies reported prolonged labor and/or obstruction. 4 studies reported fetal distress. 41 studies reported episiotomies and perineal tears. 4 studies reported pain during and after de-Female genital (anterior episiotomy) for delivery. 32 studies reported post-partum hemorrhage. 10 studies reported maternal death following FGM performed earlier in life. 10 studies reported fetal death (stillbirth and neonatal death). 3 studies reported hemorrhage antenatal at the site of FGM immediately after FGM in pregnancy. 4 studies reported antenatal infection following FGM performed in pregnancy. 1 study reported antenatal difficulty/inability to perform vaginal examination following herbs inserted to attempt to procure an abortion and subsequent vaginal atresia in pregnancy. 2 studies reported possible antenatal vesicovaginal fistula/ rectovaginal fistula following FGM in pregnancy. 1 study reported antenatal fetal injury following FGM in pregnancy. 1 study reported preterm labor following FGM in pregnancy. 1 study reported obstruction (vaginal atresia) requiring cesarean section following FGM in pregnancy. 1 study reported difficult labor following FGM in pregnancy. 2 studies reported maternal death following FGM in pregnancy. 2 studies reported fetal death following FGM in pregnancy. 1 study reported neonatal death following FGM in pregnancy	[18]
Mpinga *et al*., 2016	Medical and psychological consequences, prevalence and ethics Socio-economic consequences Direct economic consequences, school attendance, sexual and marital consequences, fertility, domestic violence, discrimination, marriageability	Concerning the research issues, 51% of the articles explored the extensive list of short- and long-term medical and psychological consequences on women, as their main research topic. 102 studies reported medical and psychological consequences after FGM. 68 studies reported prevalence and ethics after FGM. 28 studies reported socio-economic consequences after FGM. 5 studies reported direct economic consequences after FGM. 2 studies reported school attendance after FGM. 7 studies reported sexual and marital consequences after FGM. 5 studies reported fertility after FGM. 2 studies reported domestic violence after FGM. 3 studies reported discrimination after FGM. 4 studies reported marriageability after FGM.	[20]
Sripad *et al*., 2017	FGM and fistula	8 articles described the conditions as positively related the association of FGM with the occurrence of fistula. 3 articles described no association of FGM with the occurrence of fistula. 18 studies speculated that these two conditions were indirectly positively related with varying degrees of evidence.	[19]
